# Supplementary material for: Observation of helical pulses
Source: Nat Commun. 2025 Dec 8;16:10927. doi: 10.1038/s41467-025-65916-4 (PMC12686495; doi:10.1038/s41467-025-65916-4)
Supplement: Supplementary file 1 — Supplementary Information [file 41467_2025_65916_MOESM1_ESM.pdf]

Supplementary Information for:

Observation of helical pulses

Ren Wang[1,2*], Shuai Shi[1], Zeyi Zhang[1], Bing-Zhong Wang[1], Nilo Mata-Cervera[3], Miguel A. Porras[4], Yijie Shen[3,5,6*]

[1] Institute of Applied Physics, University of Electronic Science and Technology of China, Chengdu 611731, China

[2] Yangtze Delta Region Institute (Huzhou), University of Electronic Science and Technology of China, Huzhou 313098, China

[3] Centre for Disruptive Photonic Technologies, School of Physical and Mathematical Sciences, Nanyang Technological University, Singapore 637371, Singapore

[4] Grupo de Sistemas Complejos, ETSIME, Universidad Politécnica de Madrid, Rios Rosas 21, 28003 Madrid, Spain

[5] School of Electrical and Electronic Engineering, Nanyang Technological University, Singapore 639798, Singapore

[6] International Institute for Sustainability with Knotted Chiral Meta Matter (WPI-SKCM2), Hiroshima University, Hiroshima 739-8526, Japan

* E-mail: rwang@uestc.edu.cn (R.W); yijie.shen@ntu.edu.sg (Y.S.)

Supplementary Note 1. Theoretical space-time structure of quasi-linearly polarized SNHPs.

For the vector potential $\mathbf{A} = \nabla \times [f, 0, 0]$, the electromagnetic field expressions are given by:

$$\begin{cases} E(r,t) = -\mu_0 \dfrac{\partial}{\partial t} \nabla \times [1,0,0] \cdot f \\ H(r,t) = \nabla \times (\nabla \times [1,0,0] \cdot f) \end{cases} . \tag{S1}$$

Further, the individual components of the electric field can be expressed as:

$$\begin{bmatrix} E_x \\ E_y \\ E_z \end{bmatrix} = -\mu_0 \frac{\partial}{\partial t} \begin{bmatrix} 0 \\ \dfrac{\partial}{\partial z} \\ -\dfrac{\partial}{\partial y} \end{bmatrix} f . \tag{S2}$$

Thus, the electric field includes only the E_y and E_z components, with no E_x component. We focus on the remaining transverse and longitudinal components of the electric field. Supplementary Figure 1 shows the ratio of the maximum transverse electric field $\max(E_y)$ to the maximum longitudinal electric field $\max(E_z)$ at various instants of time. Overall, the electric field satisfies $\max(E_y)/\max(E_z) > 200$ at all times, indicating that this SNHP is quasi-linearly polarized.

When t=0, the spatiotemporal topology of the transversely polarized electric field component of this quasi-linearly polarized SNHP is shown in Supplementary Figure 2. The transverse electric field component exhibits a double-lobe, single-cycle helical

topology. The amplitude and phase distributions at different wavelengths are shown in Supplementary Figure 3. As seen in the figure, the SNHP consistently displays the amplitude and phase characteristics of a vortex beam with $\ell = 1$ across different wavelengths: the amplitude forms a ring-like distribution, and the phase exhibits a 360° helical pattern.

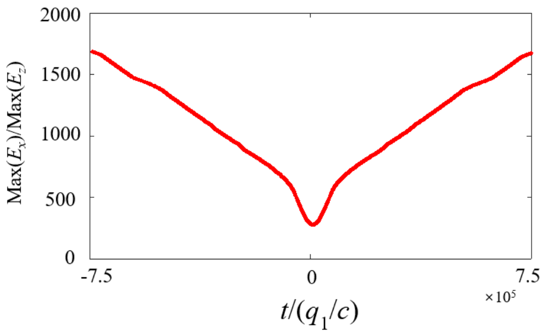

Supplementary Figure 1. The ratio of the maximum transverse electric field $\max(E_y)$ to the maximum longitudinal electric field $\max(E_z)$ over time.

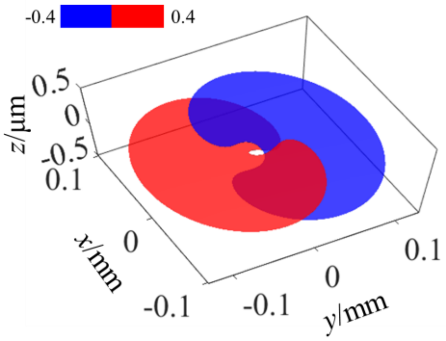

Supplementary Figure 2. Spatiotemporal topology of the transverse electric field component of quasi-linearly polarized SNHPs.

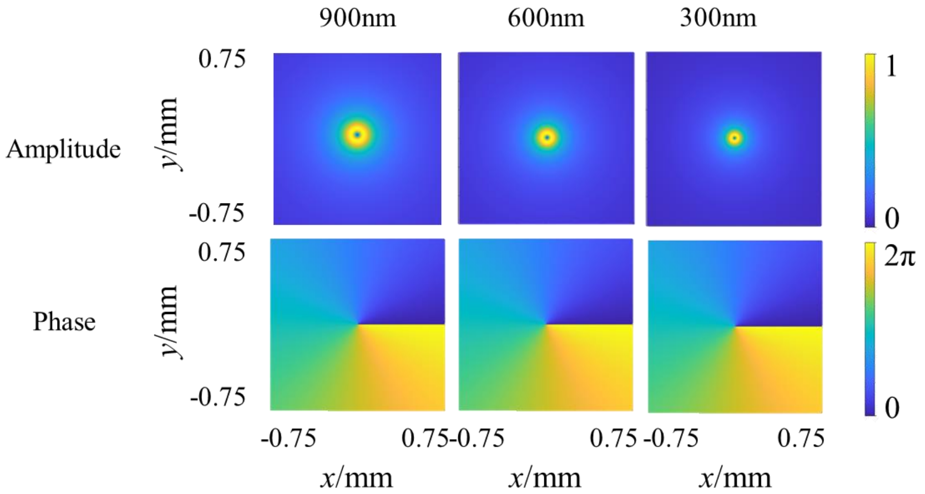

Supplementary Figure 3. Amplitude and phase distributions of the transverse electric field component of quasi-linearly polarized SNHPs at different wavelengths.

Supplementary Note 2. Theoretical space-time structure of nontransverse SNHPs.

For the vector potential $\mathbf{A} = \nabla \times [-\mathrm{i}f, f, 0]$, the electromagnetic field for nontransverse SNHPs can be obtained.

When q_1 = 0.03 m, q_2 = 20q_1, and $\ell = 1$, the spatiotemporal topologies of the transversely polarized electric field components E_x, E_y, and the longitudinally polarized electric field component E_z for the nontransverse SNHPs are shown in Supplementary Figure 4. Both transverse electric field components E_x and E_y exhibit a double-lobe, single-cycle helical topology. The longitudinal electric field component E_z displays a four-lobe helical topology. Together, these components form a nontransverse topology in the three-dimensional electric field vector of the pulses, as illustrated in Supplementary Figure 5.

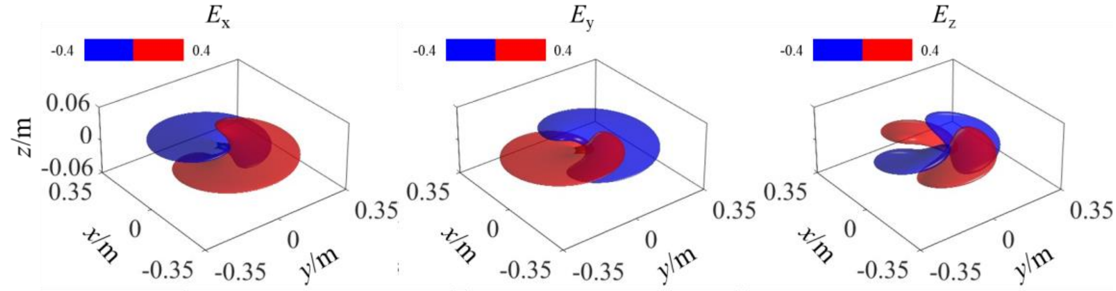

Supplementary Figure 4. Spatiotemporal topologies of the electric field components for nontransverse SNHPs.

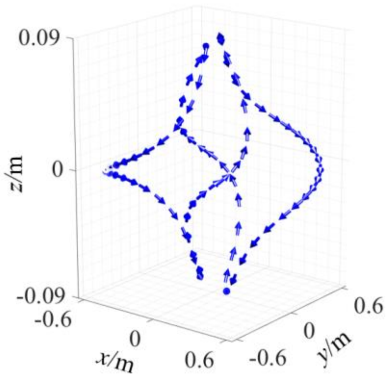

Supplementary Figure 5. Three-dimensional vector electric field of the nontransverse SNHPs.

Supplementary Note 3. Spatiotemporal field distributions of the SNHPs with $\ell = 1$ and various vector potentials.

SNHPs exhibit a range of spatiotemporal helical topologies. For consistency, the parameters for the following SNHPs are set to q_1 = 0.03 m, q_2 = 20q_1, and $\ell = 1$. Based on different vector potentials, we derived several distinct types of helical pulses in addition to the quasi-linearly polarized SNHPs and nontransverse SNHPs discussed earlier. These pulses have different field components, each with its own unique topological structure. The helical topologies of these field components may be either identical or distinct.

For example, when the vector potentials are $\mathbf{A}=\nabla\times[0,f,0]$ and $\mathbf{A}=\nabla\times[0,0,f]$, the resulting SNHPs are non-transverse pulses with E_x and E_z components and transverse pulses with E_x and E_y components, respectively, as shown in Supplementary Figure 6. When $\mathbf{A}=\nabla\times[0,f,0]$, the E_x component exhibits a single-cycle structure, while the E_z component shows a $1\frac{1}{2}$-cycle spatiotemporal vortex structure. When $\mathbf{A}=\nabla\times[0,0,f]$, both E_x and E_y components display a $1\frac{1}{2}$-cycle spatiotemporal vortex structure.

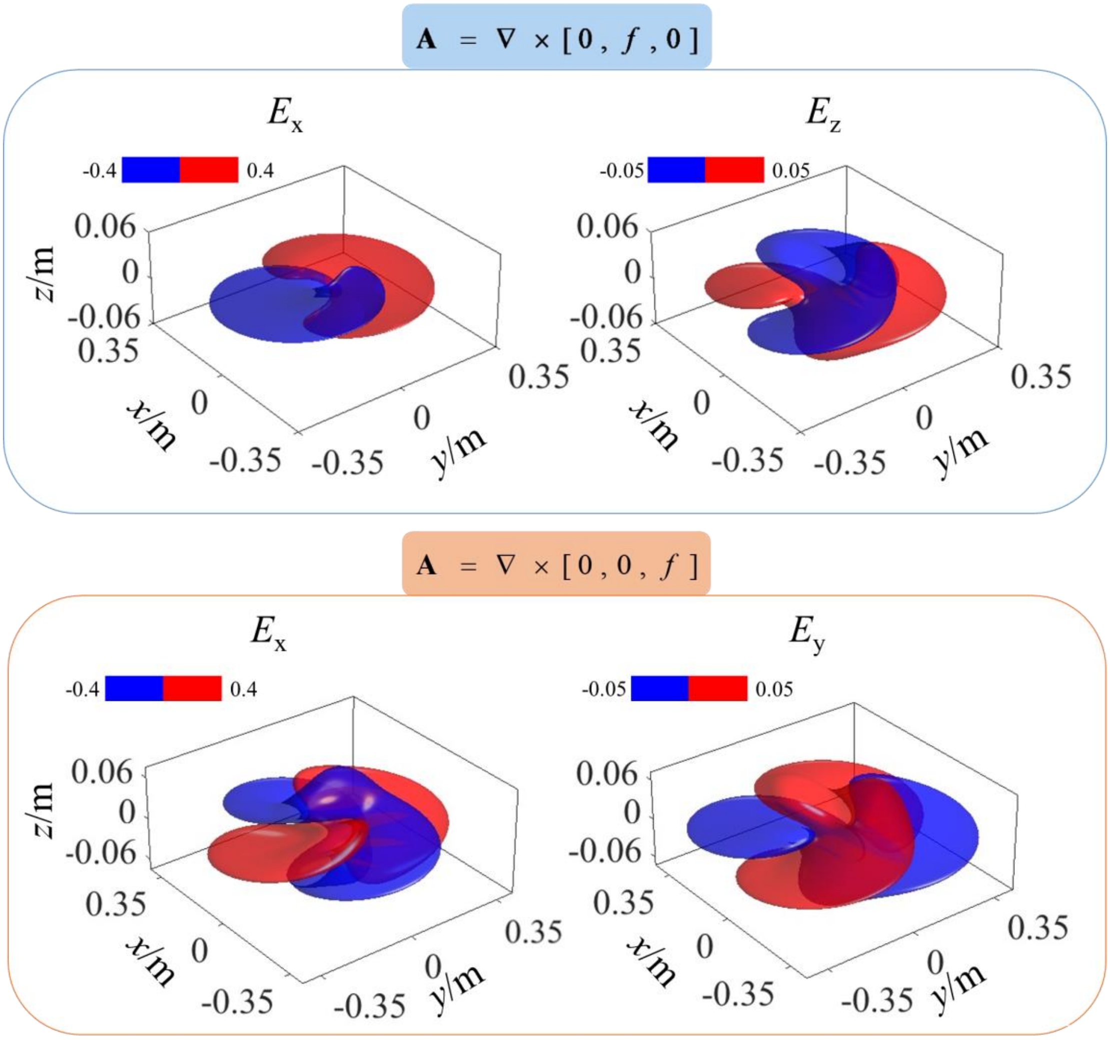

Supplementary Figure 6. Spatiotemporal topologies of SNHPs with vector potentials $\mathbf{A}=\nabla\times[0,f,0]$ and $\mathbf{A}=\nabla\times[0,0,f]$.

In addition to the number of cycles, the symmetry of different components can also be controlled by the vector potentials, resulting in some components exhibiting symmetry and others exhibiting chirality, as shown in Supplementary Figure 7. For instance, when $\mathbf{A} = \nabla \times [f, 0, 0]$, the transversely polarized components E_x and E_y of the SNHPs with superposed expressions $\mathbf{B} = \nabla \times \mathbf{A} + \mathrm{i}\partial_{ct}\mathbf{A}, \mathbf{E} = \mathrm{ci}\mathbf{B}$ are chiral, but the longitudinally polarized component E_z is not. When $\mathbf{A} = \nabla \times [0, 0, f]$, the transversely polarized electric field components, as well as the longitudinally polarized electric field component, all exhibit chirality in superposed SNHPs with $\mathbf{B} = \nabla \times \mathbf{A} + \mathrm{i}\partial_{ct}\mathbf{A}, \mathbf{E} = \mathrm{ci}\mathbf{B}$.

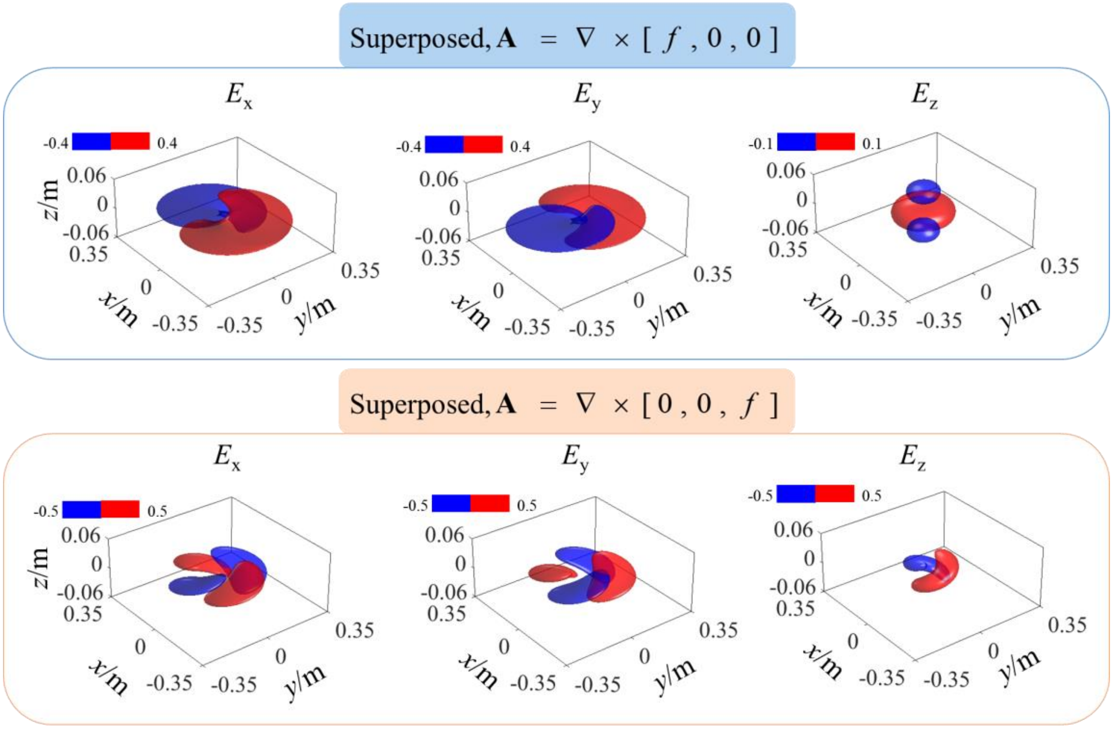

Supplementary Figure 7. Spatiotemporal topologies of various superposed SNHPs with different vector potentials.

Supplementary Note 4. Space-time nonseparability of canonical SNHPs.

Spacetime nonseparable vortex beams exhibit isodiffracting properties, meaning that

the spatial intensity distribution of each frequency component scales uniformly along the beam's trajectory across all cross-sections perpendicular to the propagation direction. To illustrate the isodiffracting nature, we define the spatially normalized intensity of monochromatic light with different wavelengths λ_i according to [47] and introduce the normalized radial position $\eta = \frac{r}{r(z)_{\max}}$, where $r(z)_{\max}$ is the location of the maximum total electric field intensity in the transverse plane at a propagation distance z. Based on these characteristics and the introduced parameters, two sets of states can be defined to describe the spacetime nonseparability exhibited by pulses [47]:

(1) Spectral States $|\lambda_i\rangle (i = 1, 2, ..., n)$: These represent monochromatic states at wavelength λ_i with peak intensity at radial position r_{λ_i}.

(2) Spatial States $|\eta_i\rangle$: These define polychromatic states located at $\eta_i = r/r_{\max}$, where $r_{\max}$ is the radial position where the total field intensity reaches its maximum.

To quantify the spacetime nonseparability of the studied vortex beams, we proceed by performing an analogy to the measurement of entanglement nonseparability, akin to that between two classical spatial fields [47]. The classical fields of the spectral state $|\lambda_i\rangle$ and the spatial state $|\eta_i\rangle$ are represented as:

$$\psi_{\lambda_i}(r,z) = \sqrt{I(\lambda_i, r, z)} H\left(r - \delta_{i-1}^{(\lambda)}\right) H\left(\delta_i^{(\lambda)} - r\right) \tag{S3}$$

$$\psi_{\eta_i}(r,z) = \sqrt{I_0(r,z)} H\left(r - \delta_{i-1}^{(\eta)}\right) H\left(\delta_i^{(\eta)} - r\right) \tag{S4}$$

where $H(r)$ is the Heaviside step function, with $H(r) = 1$ if $r > 0$ and zero otherwise. The sets of spectral and spatial states are orthogonal, $\langle \lambda_i | \lambda_j \rangle = \delta_{ij}$ and $\langle \eta_i | \eta_j \rangle = \delta_{ij}$, where δ_{ij} is the Kronecker delta. The inner product of the two states, i.e.,

the state-tomography matrix $\{c_{i,j}\}$, is given by $\langle \eta_i | \lambda_j \rangle = \int \varepsilon_{\eta_i} \varepsilon^*_{\lambda_j} dr$.

According to the definitions of spectral and spatial states, the tomography matrix for an isodiffracting pulse should be diagonal. The results are shown in Supplementary Figure 7. From the evaluated state-tomography matrix, we can reconstruct the corresponding density matrix of the spatial-spectral states, $\tilde{\xi} = |\tilde{\psi}\rangle|\tilde{\psi}\rangle$ (where $\tilde{\psi}$ is the state). Notably, knowledge of the density matrix allows us to apply quantum measurements, such as fidelity, concurrence, and entanglement of formation, to quantitatively characterize the pulse's properties.

(1) Fidelity: Here, we set the target state as the ideal spacetime nonseparable vortex beam $|\psi\rangle = \sum_{i=1}^{n} c_i |r_i\rangle|\lambda_i\rangle$. The fidelity of the measured state can then be calculated as $F = \langle \tilde{\psi} | \tilde{\zeta} | \tilde{\psi} \rangle$, where $\tilde{\zeta}$ is the density matrix of the measured state. Fidelity quantitatively measures the similarity between the pulse under study and the ideal spacetime nonseparable vortex beam, ranging from 0 to 1, with higher values indicating greater similarity to the ideal target state.

(2) Concurrence (Con): defined as $con = \sqrt{2\left[1 - Tr\left(\rho_A^2\right)\right]} \Big/ \sqrt{2[1 - 1/n]}$, where ρ_A is the reduced density matrix. For any n-dimensional state, the normalized concurrence ranges from 0 to 1, indicating no entanglement (or complete separability) and strong nonseparability (maximal entanglement), respectively.

(3) Entanglement of Formation (EoF): calculated from the reduced density matrix as $EoF = -Tr\left[\rho_A \log_2(\rho_A)\right] / \log_2(n)$ in n-dimensional cases. A higher EoF for a spacetime nonseparable beam indicates stronger spacetime nonseparability.

The state-tomography matrix and state density matrix of the transversely polarized

electric field component E_x of the quasi-linearly polarized SNHPs are presented in Supplementary Figure 8. The diagonalization of the state-tomography matrix and the uniformity of the state density matrix indicate a strong degree of spatiotemporal nonseparability. This spatiotemporal nonseparability is also visually apparent in the propagation trajectories of individual components of the spatiotemporal pulses. The trajectories of different frequency components during the propagation of the quasi-linearly polarized SNHPs are shown in Supplementary Figure 9. It is evident from the figure that the trajectories of various frequency components do not intersect, confirming the isodiffracting characteristics. This observation is consistent with the trends in *Con* and *EoF* depicted in Supplementary Figure 10. The con and EoF values corresponding to the transverse electric field component E_x of the quasi-linearly polarized SNHPs remain close to 1 throughout the propagation distance.

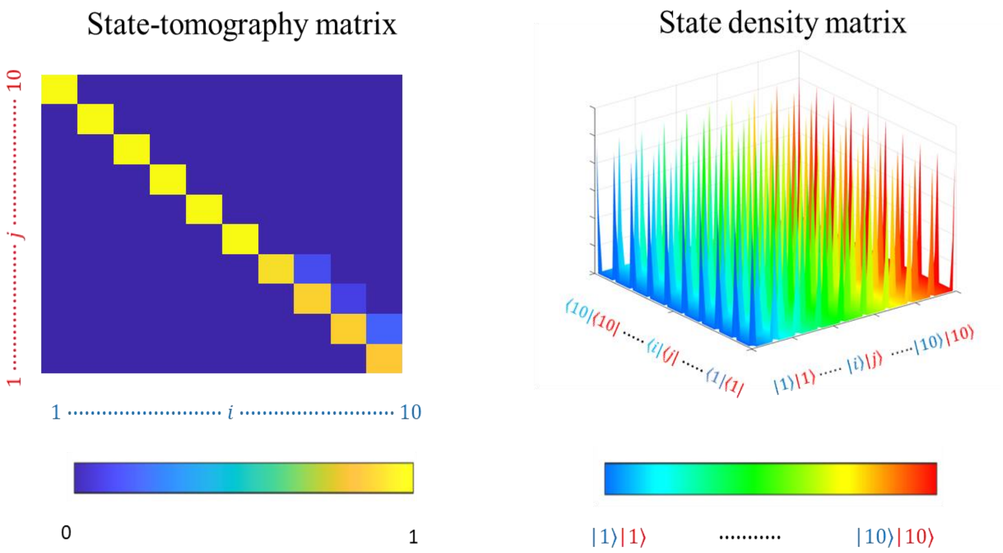

Supplementary Figure 8. State-tomography matrix and state density matrix of the transversely polarized electric field component E_x of the quasi-linearly polarized SNHPs.

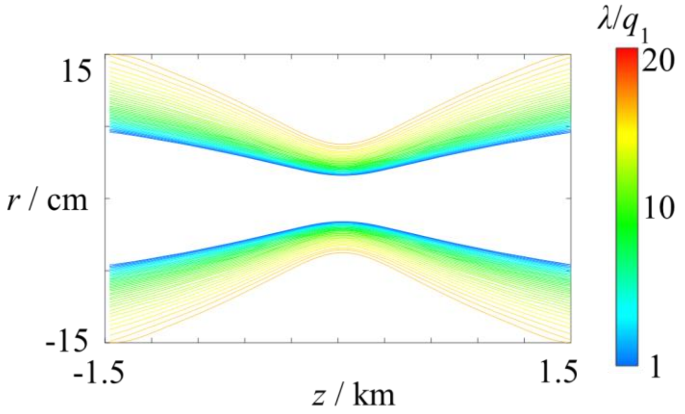

Supplementary Figure 9. Trajectories of different frequency components during the propagation of the transversely polarized electric field component E_x of the quasi-linearly polarized SNHPs.

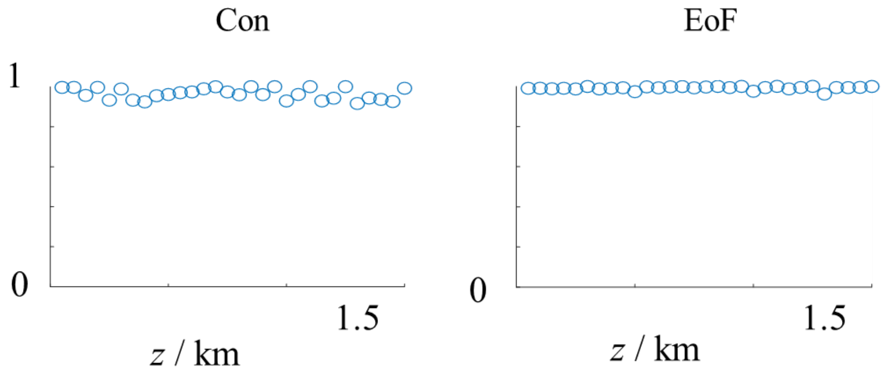

Supplementary Figure 10. Concurrence (Con) and entanglement of formation (EoF) of the transversely polarized electric field component E_x of the quasi-linearly polarized SNHPs versus propagation distance.

The state-tomography matrix and state density matrix of the transversely polarized electric field components E_x and E_y of the nontransverse SNHPs are shown in Supplementary Figure 11. Similar to the quasi-linearly polarized SNHPs, the state-tomography matrix is diagonal, and the state density matrix is uniform, indicating

strong spatiotemporal nonseparability. The propagation trajectories of different frequency components in the nontransverse SNHPs are illustrated in Supplementary Figure 12. As shown, the trajectories of various frequency components do not intersect, confirming the isodiffracting nature of these pulses. This observation aligns with the trends in con and EoF depicted in Supplementary Figure 13. The con and EoF values associated with the transverse electric field components E_x and E_y of the nontransverse SNHPs remain close to 1 throughout the propagation distance. Therefore, both the quasi-linearly polarized SNHPs and the nontransverse SNHPs exhibit strong spatiotemporal nonseparability.

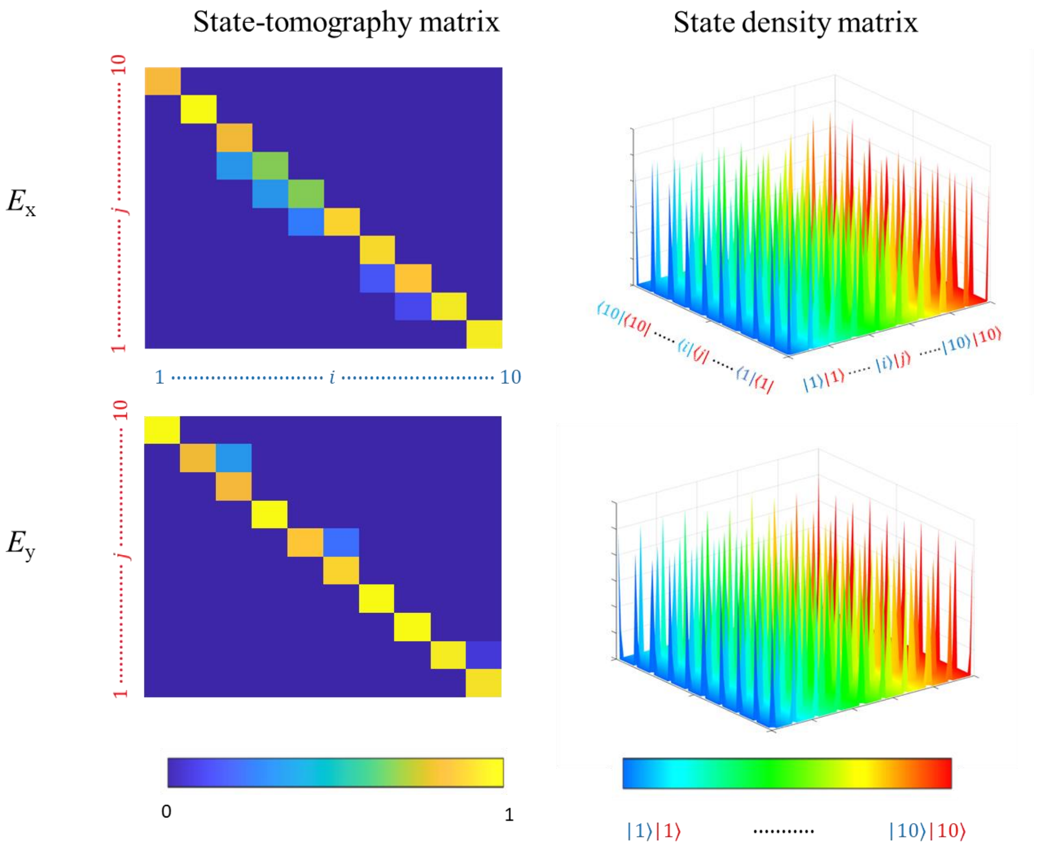

Supplementary Figure 11. State-tomography matrix and state density matrix of the transversely polarized electric field components E_x and E_y of the nontransverse SNHPs.

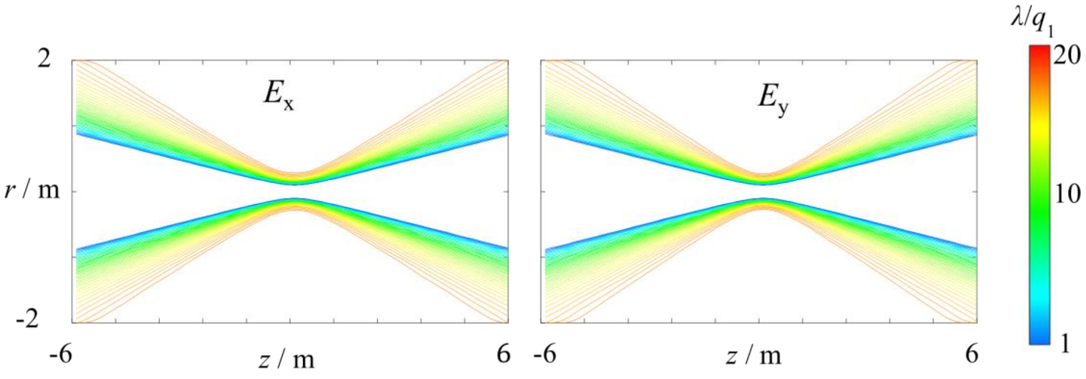

Supplementary Figure 12. Trajectories of different frequency components during the propagation of the transversely polarized electric field components E_x and E_y of the nontransverse SNHPs.

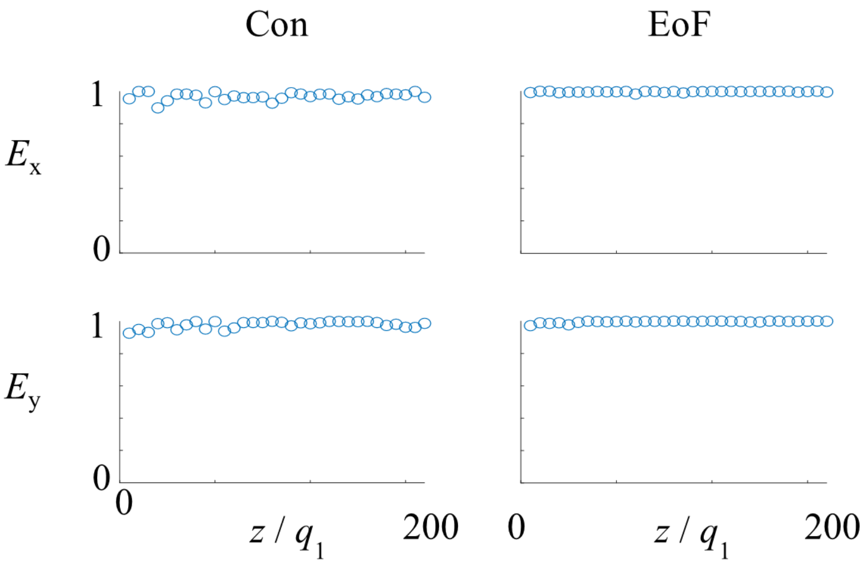

Supplementary Figure 13. Con and EoF of the transversely polarized electric field components E_x and E_y of the nontransverse SNHPs versus propagation distance.

Supplementary Note 5. Performances discussions of the generation scheme for quasi-linearly polarized optical SNHPs.

Toroidal pulses exhibit a broad spectral distribution, consisting of a superposition of

various wavelengths, as illustrated in Supplementary Figure 14 and Methods in the main text. Consequently, we decomposed the fields into different wavelength components using a filtering system, as depicted in Supplementary Figure 14(b). We utilized TM optical toroidal pulses with q_1=192 nm and q_2=75000q_1 reported in [52] as the input light and employed a quarter-wave plate (QWP) and a polarizer to achieve circular and linear polarization decomposition, respectively. Subsequently, we analyzed the vortex nature of the generated waves through edge diffraction patterns.

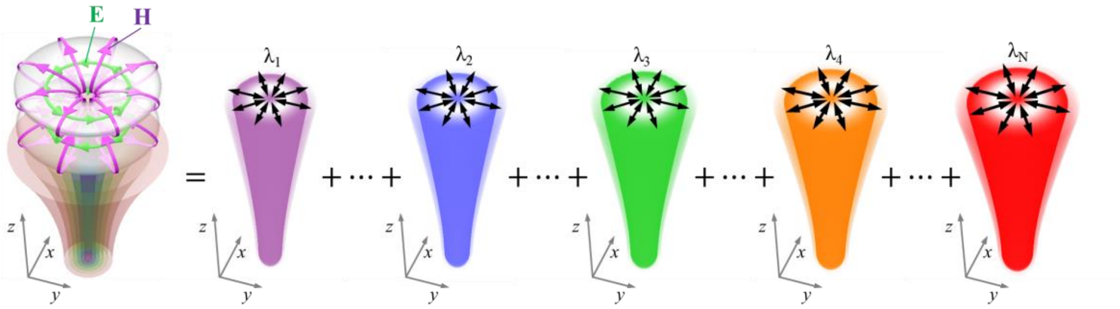

Supplementary Figure 14. Decomposition of the fields of toroidal pulses. The toroidal pulse is decomposed into distinct wavelength components for analysis.

When polarization decomposition waveplates were not used, the diffraction patterns of TM optical toroidal pulses with q_1=192 nm and q_2=75000q_1, as observed by the experimental system, are shown in Supplementary Figure 15. Without an opaque edge, the CCD detected the light intensity distribution at different wavelengths, revealing ring-shaped intensity distributions across all wavelengths. In the presence of an opaque edge, the diffraction patterns did not exhibit distinct fork-shaped patterns at each wavelength, indicating the absence of optical vortices.

Changing the rotation direction of the circular polarizer allows for the generation of

SNHPs with opposite chirality. The diffraction patterns of the generated left-handed chiral SNHPs are shown in Supplementary Figure 16. In the absence of an opaque edge, the intensities displayed ring-shaped distributions across all wavelengths, consistent with the intensity distribution of SNHPs. With an opaque edge present, the diffraction patterns observed by the experimental system showed distinct fork-shaped patterns at each wavelength, indicating the presence of optical vortices consistent with those of SNHPs.

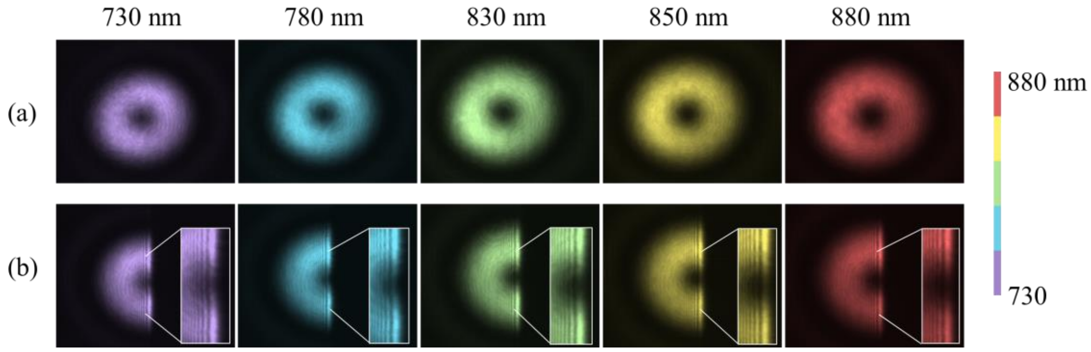

Supplementary Figure 15. Diffraction patterns of TM optical toroidal pulses. The intensity distributions of light detected by CCD camera at different wavelengths in the absence and presence of an opaque edge are shown in (a) and (b), respectively.

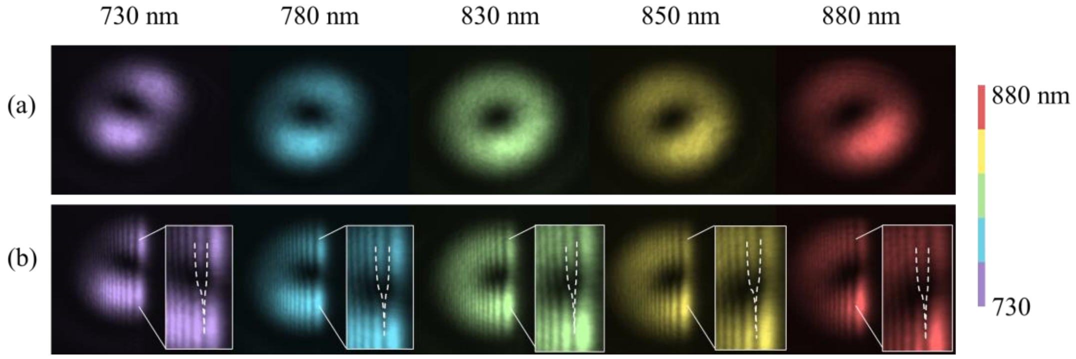

Supplementary Figure 16. Diffraction patterns of generated left-handed chiral SNHPs. The intensity distributions of light detected by CCD camera at different wavelengths in the absence and presence of an opaque edge are shown in (a) and (b), respectively.

The spacetime nonseparability of SNHPs is inherited from the incident optical toroidal pulses. As a comparison, when the incident wave is a radially polarized Gaussian beam, the tracking curves of the maximum field positions for different wavelengths of optical pulses generated are shown in Supplementary Figure 17. As observed from the figure, when the incident wave is a radially polarized Gaussian beam, even with the use of a quarter-wave plate (QWP) and a polarizer to achieve circular and linear polarization decomposition, the spectral tracking curves of the generated pulses still exhibit crossing behavior, indicating poor spacetime nonseparability and a poor match with canonical SNHPs. In addition, when the incident wave is a radially polarized Gaussian beam, the non-diagonalized matrix visually demonstrates non-isodiffraction characteristics. The measured fidelity is only 0.0657, indicating a poor match with canonical SNHPs.

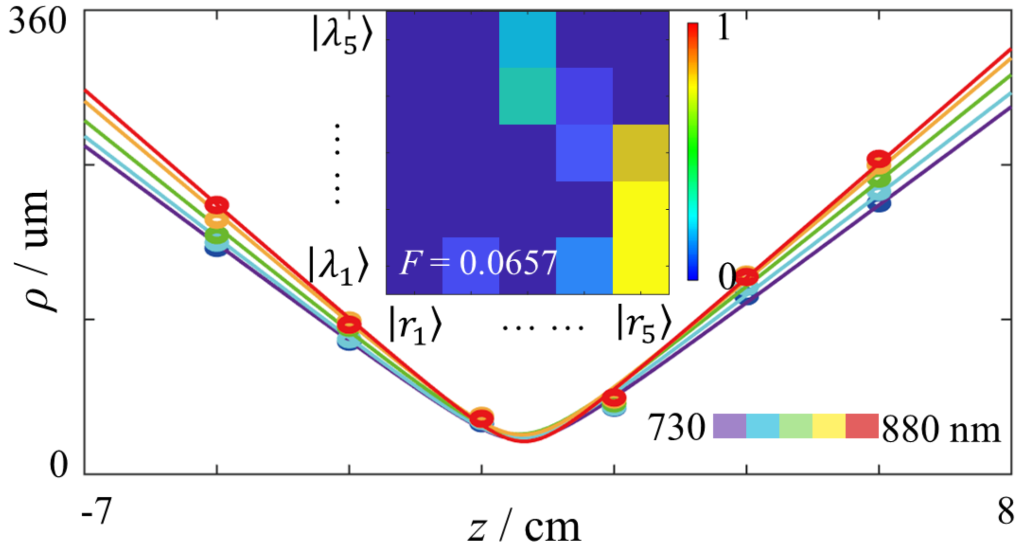

Supplementary Figure 17. Measured tracking curves of the maximum field positions for different wavelengths when the incident wave is a radially polarized Gaussian beam. The inserted figure shows the state-tomography matrix of generated pulses when a

radially polarized Gaussian beam. The non-diagonalized matrix visually demonstrates non-isodiffraction characteristics. The measured fidelity (F) is only 0.0657, indicating a poor match with canonical SNHPs.

Supplementary Note 6. Analysis of the input toroidal pulses.

The toroidal field structures were verified prior to decomposition using the setup shown in Supplementary Figure 2 of [52]. This setup is based on a Ti:Sapphire laser, which, in combination with a pulse shaper (Biophotonics MIIPS Box640), generates 10 fs pulses centered at ~800 nm with a bandwidth of approximately 200 nm.

The verified toroidal field structures are shown in Supplementary Figure 18. The E_ρ component of the canonical toroidal pulse, with parameters q_1=192 nm and q_2=75000q_1, exhibits a single-cycle structure (Supplementary Figure 18(a)). Its spectrum, shown in Supplementary Figure 19, spans a broad range over 400–2000 nm, significantly wider than the operating wavelength range of the employed laser (700–900 nm). When the spectrum is truncated to the 700–900 nm range and the corresponding spatiotemporal field is reconstructed via inverse Fourier transform, the resulting field (Supplementary Figure 18(b)) exhibits a few-cycle structure. This structure is consistent with the measured E_ρ component of the input toroidal pulse (Supplementary Figure 18(c)) and aligns with the findings reported in [52]. This analysis confirms that a laser with a bandwidth covering only a portion of the toroidal pulse spectrum can generate a few-cycle toroidal pulse. As shown in the main text, inputting such a few-cycle toroidal pulse produces SNHPs that also exhibit few-cycle

characteristics. Due to bandwidth limitations in the optical regime, we cannot generate optical single-cycle toroidal pulses or single-cycle SNHPs at present. Therefore, as a complement, we have experimentally demonstrated the generation of single-cycle SNHPs in the microwave regime.

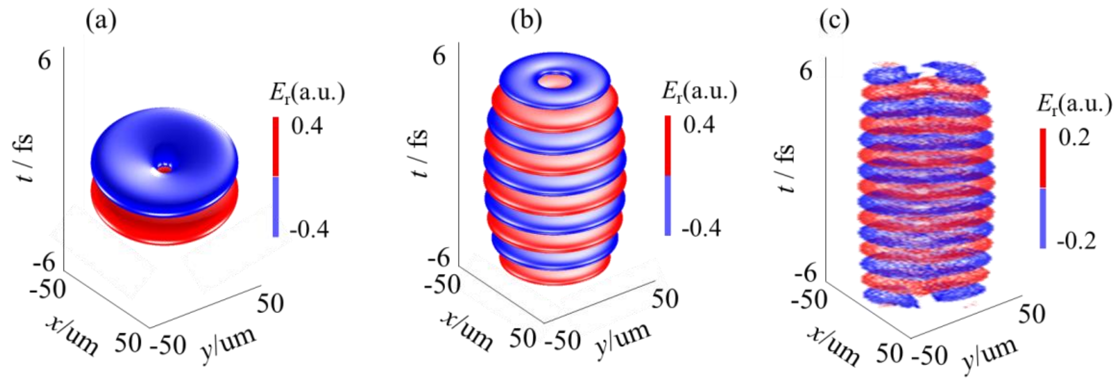

Supplementary Figure 18. Transversely polarized component of the toroidal pulse with parameters q_1=192 nm and q_2=75000q_1. (a) The canonical toroidal pulse, exhibiting a single-cycle structure; (b) The toroidal pulse reconstructed from a partial spectrum, exhibiting a few-cycle structure; (c) Measured E_ρ component of the input toroidal pulse, showing a few-cycle structure consistent with (b). The red and blue regions correspond to electric fields with opposite phases.

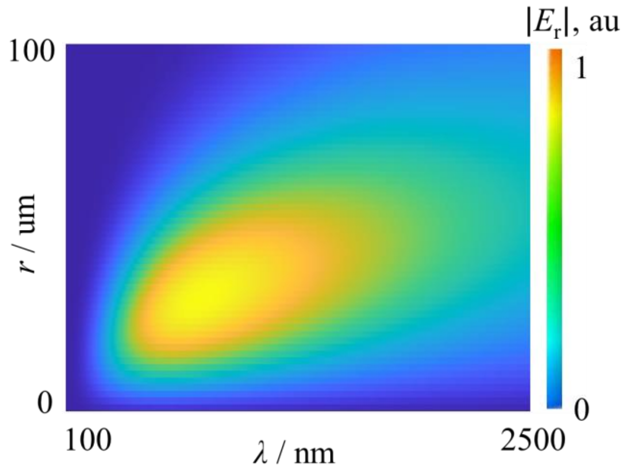

Supplementary Figure 19. Spectrum of the toroidal pulse with parameters q_1=192 nm

and q_2=75000q_1. The main spectral range spans 400–2000 nm, which is significantly broader than the operating wavelength range of the employed laser (700–900 nm).

Supplementary Note 7. Spiral emitter for the generation of nontransverse SNHPs.

The nontransverse SNHPs in this paper were generated using a dual-arm Archimedean spiral emitter, as illustrated in Supplementary Figure 20. The antenna comprises three main components: two radiating arms fed in-phase, a dielectric substrate, and a feed structure. The radiating arms follow the parametric equation of an Archimedean spiral:

$$\begin{cases} X_1 = (a+bt)\cos(2\pi nt) \\ Y_1 = (a+bt)\sin(2\pi nt) \\ X_2 = (a+bt)\cos(\theta+2\pi nt) \\ Y_2 = (a+bt)\sin(\theta+2\pi nt) \end{cases} \quad \text{(S5)}$$

In our design, the parameters are set to $a=0, b=25, n=25$, and the duty cycle factor $\theta=\pi/3$. The structure of the dual-arm spiral is shown in Supplementary Figure 20. The uppermost layer of the spiral emitter consists of a metal dual-arm spiral, which serves as the radiating element. This metal layer is etched from a metal plate to form the spiral. The middle layer is a dielectric disk made of FR4, with a dielectric constant ε_r=4.4. The disk has a radius of 60 mm and a thickness of 1 mm. The in-phase feeding of the two radiating arms is achieved using a coaxial probe with an inner conductor radius of 1.28 mm. The inner conductor of the coaxial line connects to the starting points of the dual-arm spiral through a via in the substrate, while the outer conductor remains isolated.

The reflection coefficient of the emitter, as measured, is shown in Supplementary

Figure 21. Across the range of 1 GHz to 9 GHz, the reflection coefficient remains around -10 dB, indicating significant broadband characteristics of the dual-arm Archimedean spiral emitter, which effectively covers most of the target SNHPs' frequency range.

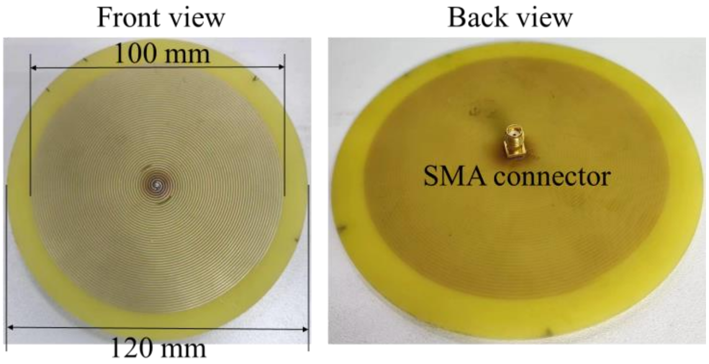

Supplementary Figure 20. Pictures of the dual-arm Archimedean spiral emitter.

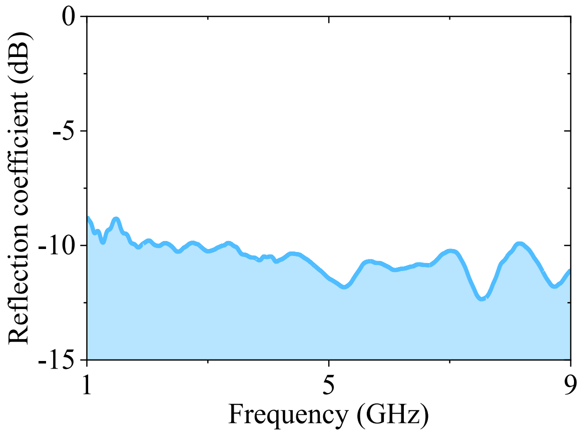

Supplementary Figure 21. Measured reflection coefficient of the dual-arm Archimedean spiral emitter.

Supplementary Note 8. Transversely polarized component measurement of nontransverse SNHPs.

The measurement setup for the transversely polarized component of the nontransverse SNHPs is shown in Supplementary Figure 22. We used an R&S® ZNA vector network analyzer to measure the S_{21} parameter of the spiral emitter as the spatial channel response. The vector network analyzer supports a testing range of 10 MHz to 50 GHz. The receiving antenna is a waveguide probe, and due to the operational band and mode of the waveguide antenna, we conducted tests across four waveguide bands: $1.7-2.8\text{GHz}$, $2.8-3.95\text{GHz}$, $3.95-5.85\text{GHz}$, and $5.85-8.2\text{GHz}$.

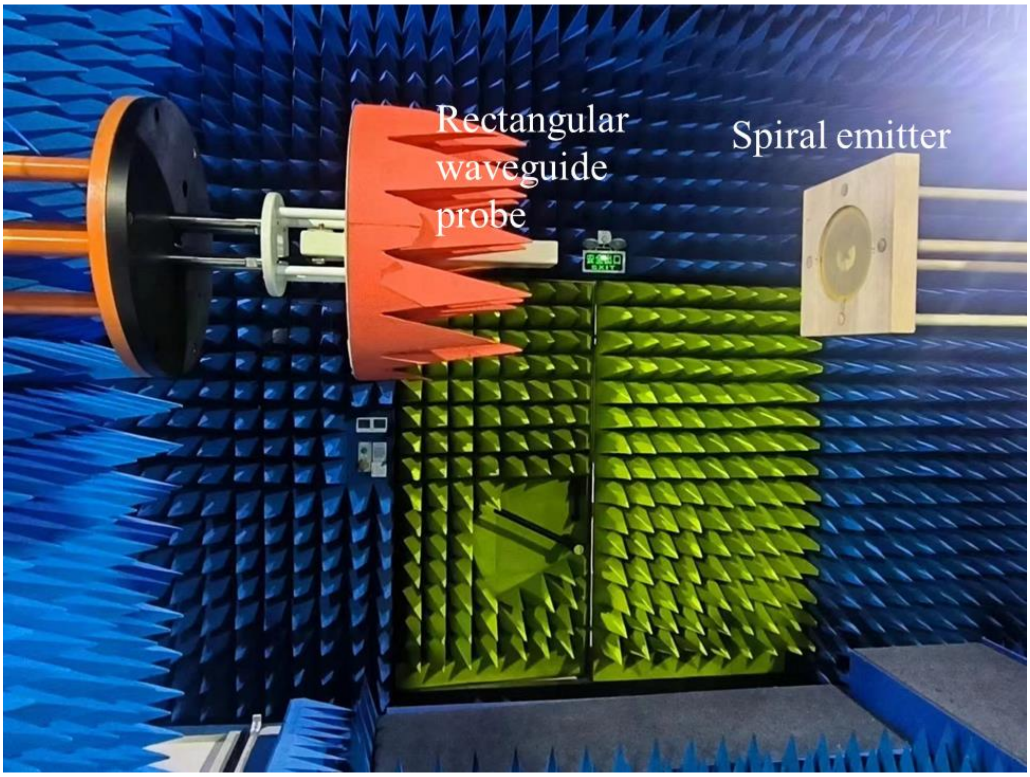

Supplementary Figure 22. Measurement setup for the transversely polarized component of nontransverse SNHPs.

We conducted measurements across the four waveguide-covered bands with a frequency step of 50 MHz, measuring the two transverse components separately. The measurement environment allows for flexible movement of the receiving antenna

within the measurement space. Supplementary Figure 23 shows the amplitude and phase distributions of the two transverse components at 2 GHz, 4 GHz, 6 GHz, and 8 GHz at a position 0.4 m above the antenna plane. At each frequency, the amplitude exhibits a distinct hollow ring structure, and the phase shows a clear 360-degree vortex phase distribution.

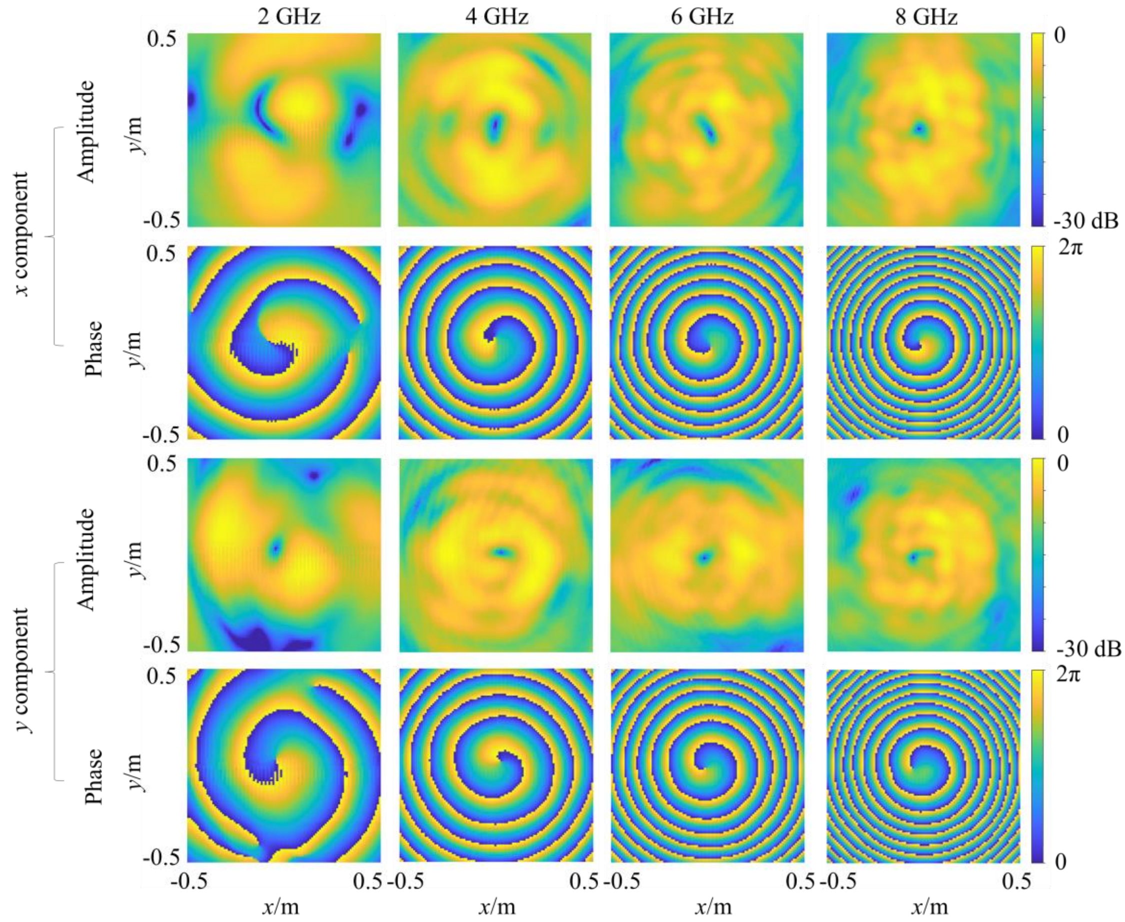

Supplementary Figure 23. Measured amplitude and phase distributions at several frequencies for the transversely polarized component of nontransverse SNHPs.

The simulated and measured spatial spectrum distributions of the E_x component of the nontransverse SNHPs are presented in Supplementary Figure 24. Both the simulated and measured SNHPs demonstrate a wide bandwidth, with the spatial

spectrum narrowing as the frequency increases and the maximum moving closer to the central axis ρ=0. This behavior is consistent with that of the canonical SNHPs and aligns with the spatial spectrum variations of the E_y component shown in the main text.

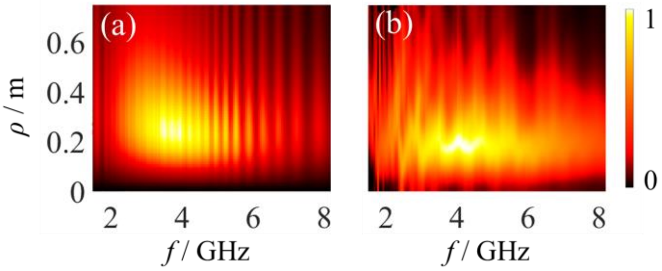

Supplementary Figure 24. (a) Simulated and (b) measured spatial spectrum distribution of the E_x component of nontransverse SNHPs.

Supplementary Note 9. Longitudinally polarized component measurement of nontransverse SNHPs.

We used a monopole antenna to probe the longitudinally polarized component of nontransverse SNHPs. The monopole antenna operates within a frequency range of 1.4 – 10.5 GHz, covering the required measurement band. The measurement setup is shown in Supplementary Figure 25, with other conditions identical to those used for measuring the transverse components.

Supplementary Figure 26 presents the corrected amplitude and phase distributions of the longitudinal component at 2 GHz, 4 GHz, 6 GHz, and 8 GHz, measured 0.4 m from the spiral emitter plane. At each frequency, the amplitude exhibits a clear hollow ring structure, while the phase displays a distinct 720-degree vortex pattern. The spatial spectrum distributions of the longitudinally polarized component E_z of nontransverse

SNHPs, both simulated and measured, are shown in Supplementary Figure 27. Both the simulations and measurements reveal a wide bandwidth, with the spatial spectrum narrowing as the frequency increases and the maximum shifting toward the central axis ρ=0. Despite some fluctuations in the measured spectrum due to testing errors, the overall trend aligns well with the simulations.

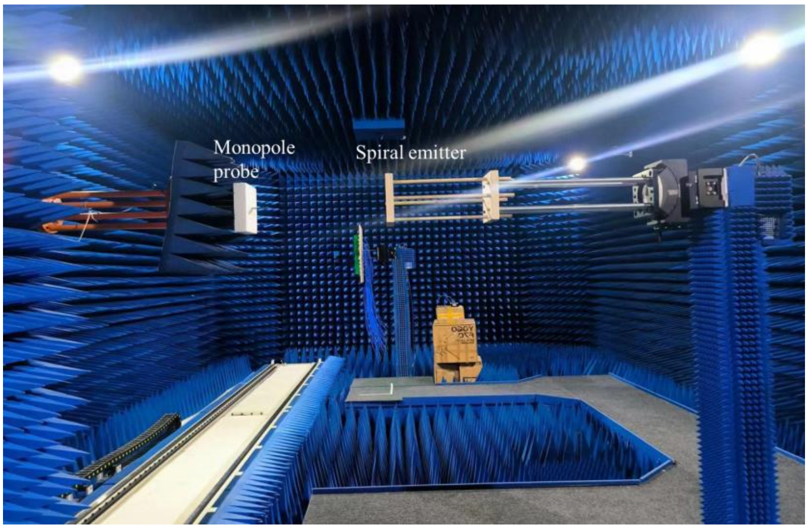

Supplementary Figure 25. Measurement setup for the longitudinally polarized component of nontransverse SNHPs.

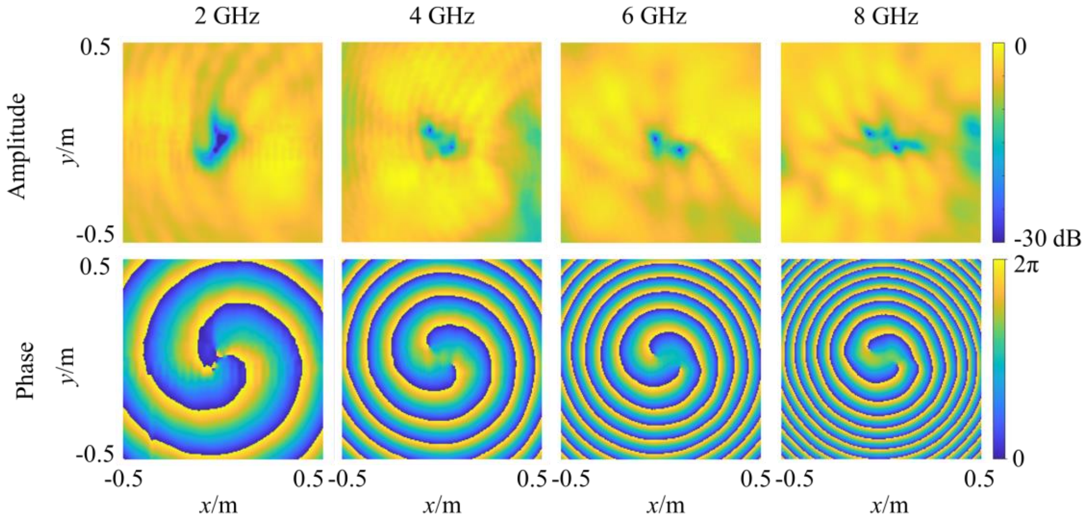

Supplementary Figure 26. Measured amplitude and phase distributions at several frequencies for the longitudinally polarized component of nontransverse SNHPs.

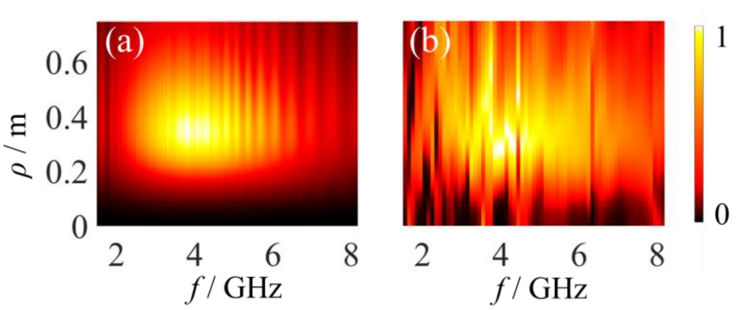

Supplementary Figure 27. (a) Simulated and (b) measured spatial spectrum distribution of the longitudinally polarized component of nontransverse SNHPs.

Supplementary Note 10. Spacetime field construction for nontransverse SNHPs.

Our goal is to generate SNHPs with a spatiotemporal vortex morphology and a specific spectral state, represented by the target spacetime field $y_{\text{idel}}(\omega,r)$. This spacetime field can be considered as the result of the excitation signal and the spatial response of the spiral emitter.

Once the spatial response $h(\omega,r)$ of the spiral emitter is obtained through simulation or measurement, the input signal $x(\omega)$ can be adjusted to achieve the desired variation in the radiated spatial field $y(\omega,r)$. To better match the target SNHPs spacetime field, we select the signal at the target field location r_1 as the target signal $y_{\text{idel}}\left(\omega,r_1^{\text{idel}}\right)$, setting $y\left(\omega,r_1\right)=y_{\text{idel}}\left(\omega,r_1^{\text{idel}}\right)$. Using the spatial response $h(\omega,r)$ of the spiral emitter, we then calculate the input signal $x(\omega)=y_{\text{idel}}\left(\omega,r_1^{\text{idel}}\right)/\,h\left(\omega,r_1\right)$ that ensures the field generated at r_1 aligns with the ideal field. By optimizing the location of r_1, we can construct a generated field that best matches the canonical SNHPs, thereby obtaining the optimal excitation signal.

The spacetime field structures of the E_x component of the generated nontransverse

SNHPs, obtained through this spacetime field construction method, are shown in Supplementary Figure 28. Both the simulated and measured E_x components exhibit a double-lobe, single-cycle helical topology similar to that of the canonical SNHPs. The spatiotemporal field distribution of E_x resembles the single-cycle helical topology observed in the E_y component distributions shown in Figs. 3(b1-b3) of the main manuscript.

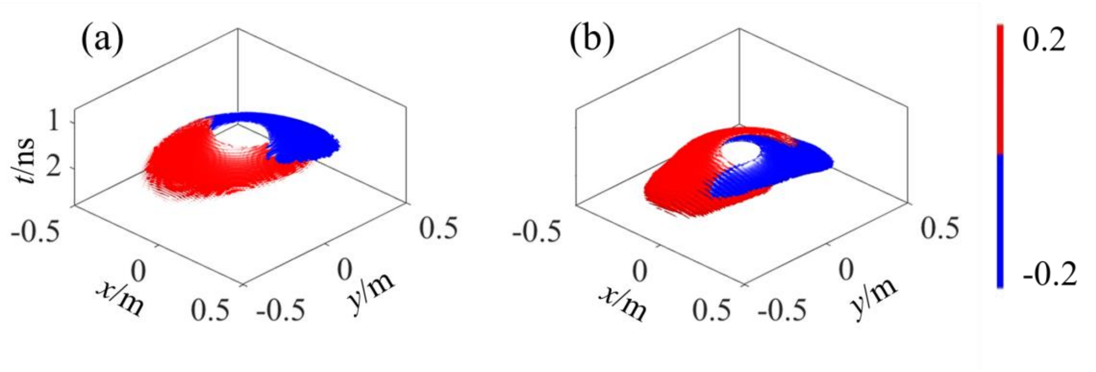

Supplementary Figure 28. (a) Simulated and (b) measured spatiotemporal waveforms of the E_x component of the generated nontransverse SNHPs.

Supplementary Note 11. State-tomography matrices and state density matrices of generated nontransverse SNHPs.

The tracking curves of the maximum field positions for different wavelengths of pulses generated by the spiral emitter are shown in Supplementary Figure 29. The trajectories of different wavelengths do not cross, demonstrating isodiffraction characteristics.

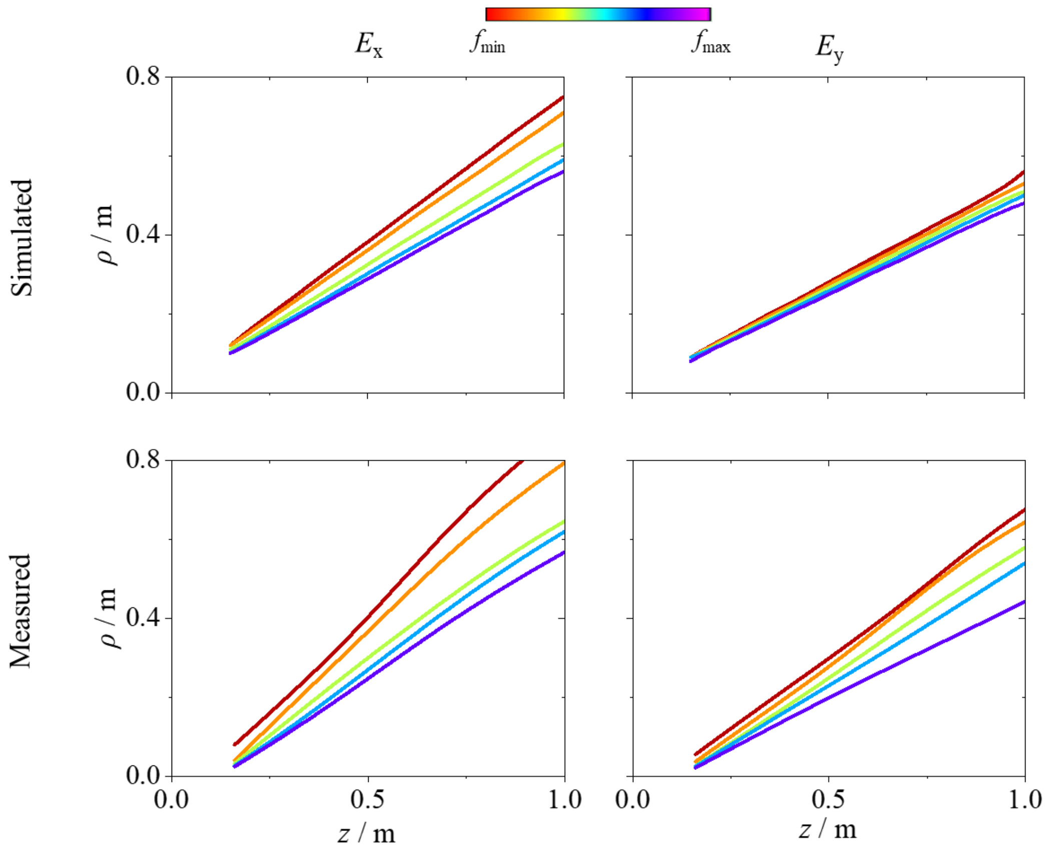

Supplementary Figure 29. Tracking curves of the simulated and measured maximum field positions for different wavelengths of the transversely polarized electric field components E_x and E_y of the generated nontransverse SNHPs.

The simulated state-tomography matrix and state density matrix of the transversely polarized electric field components E_x and E_y of the generated nontransverse SNHPs are shown in Supplementary Figure 30. The simulated state-tomography matrices are nearly diagonal, and the state density matrices are approximately uniform, indicating good spatiotemporal nonseparability. The consistency between simulation and experimental results aligns with the Con and EoF values close to 1 reported in the main text. The simulated fidelities exceed 0.98, suggesting a perfect match with canonical SNHPs.

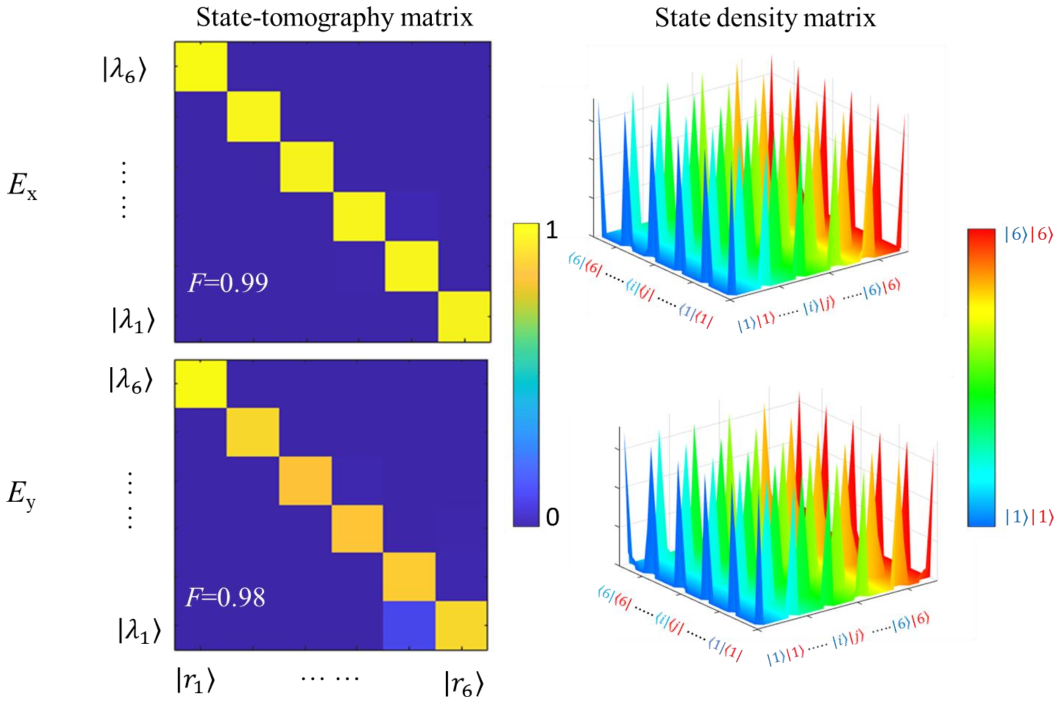

Supplementary Figure 30. Simulated state-tomography matrix and state density matrix of the transversely polarized electric field components E_x and E_y of the generated nontransverse SNHPs. *F* denotes fidelity.

Supplementary Note 12. Performances of spiral emitter with ground for the generation of nontransverse SNHPs.

Typically, microwave spiral antennas incorporate a ground plane on the backside to achieve unidirectional radiation, which makes it challenging to observe SNHPs. Ground reflections would disrupt the structure of SNHPs, particularly their spacetime nonseparability. The simulated state-tomography matrix and state density matrix of the transversely polarized electric field components E_x and E_y of pulses generated by a spiral emitter with a ground plane are shown in Supplementary Figure 31. Compared to the performance of the spiral emitter without a ground plane, as shown in

Supplementary Figure 30, the state-tomography and state density matrices appear disordered, indicating poor spatiotemporal nonseparability. The simulated fidelities of the pulses generated by the spiral emitter with a ground plane are only approximately 0.1, signifying poor spacetime nonseparability and a poor match with canonical SNHPs. Therefore, the absence of a ground plane on the backside of the spiral emitter's substrate is crucial for generating SNHPs.

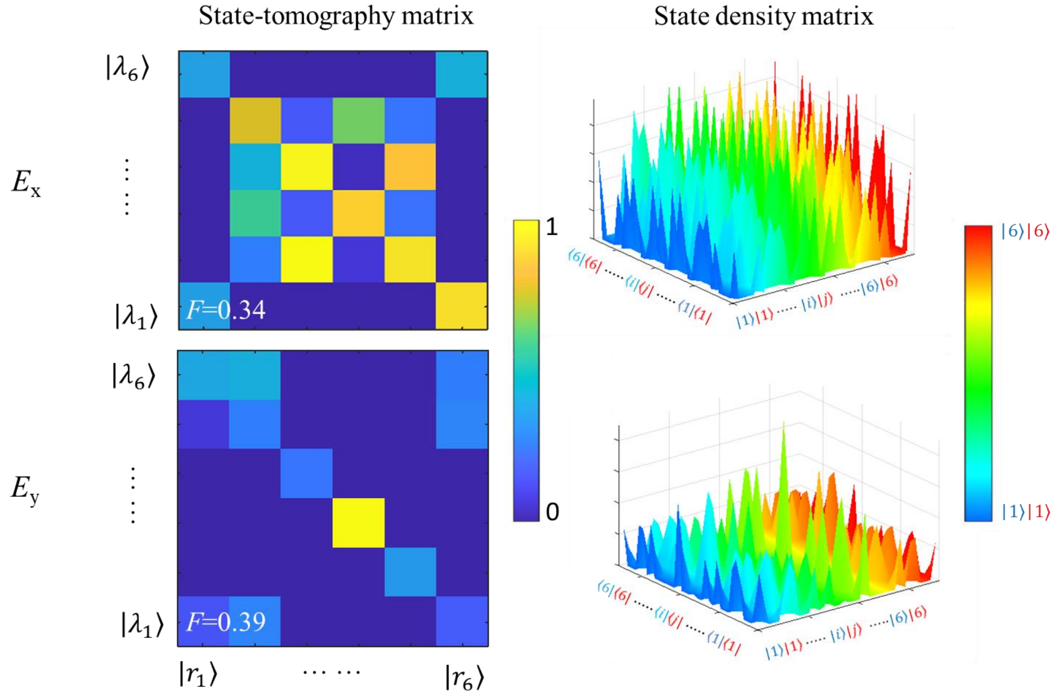

Supplementary Figure 31. Simulated state-tomography matrix and state density matrix of the transversely polarized electric field components E_x and E_y of the pulses generated by a spiral emitter with ground. F denotes fidelity.

Supplementary Note 13. Comparison between SNHPs and spatiotemporal optical vortices (STOVs).

Based on the observed results, SNHPs constitute a new class of space–time

structured pulses that are distinct from STOVs in several key aspects. A brief comparison between SNHPs and STOVs is shown in Supplementary Figure 32. The main differences between SNHPs and STOVs are summarized as follows:

1. Although both STOVs and SNHPs exhibit vortex characteristics, the vortex structure of SNHPs is manifested in the three-dimensional spatiotemporal field distribution (Supplementary Figures 32(a2)–(a3)), whereas that of STOVs appears in the phase singularity of a longitudinal cross-section (Supplementary Figure 32(b1)), rather than in the full 3D spatiotemporal field (Supplementary Figure 32(a1)).
2. SNHPs feature a helical topology and wavefront (Supplementary Figures 32(a2)–(a3)), while STOVs exhibit a toroidal topology (Supplementary Figure 32(a1)) accompanied by a planar wavefront (Supplementary Figure 32(b1)).
3. SNHPs are single-cycle or few-cycle ultra-wideband pulses (Supplementary Figures 32(b2)–(b3)), with a wide bandwidth (exceeding 120% in Supplementary Figures 32(c2)–(c3)), whereas STOVs are multi-cycle (Supplementary Figure 32(b1)), narrowband quasi-monochromatic pulses (with a bandwidth of approximately 10% in Supplementary Figure 32(c1)).
4. The spectral profiles of SNHPs and STOVs are distinct. The spatial–spectral distribution of a STOV features a tilted nodal line (Supplementary Figure 32(c1)), whereas that of an SNHP exhibits a triangular-shaped dominant spectral region (Supplementary Figures 32(c2)–(c3)).
5. Some SNHPs are non-transverse waves, as shown in Supplementary Figures

32(a2)–(a3), (b2)–(b3), and (c2)–(c3), while STOVs are purely transverse waves (Supplementary Figures 32(a1), (b1), and (c1)).

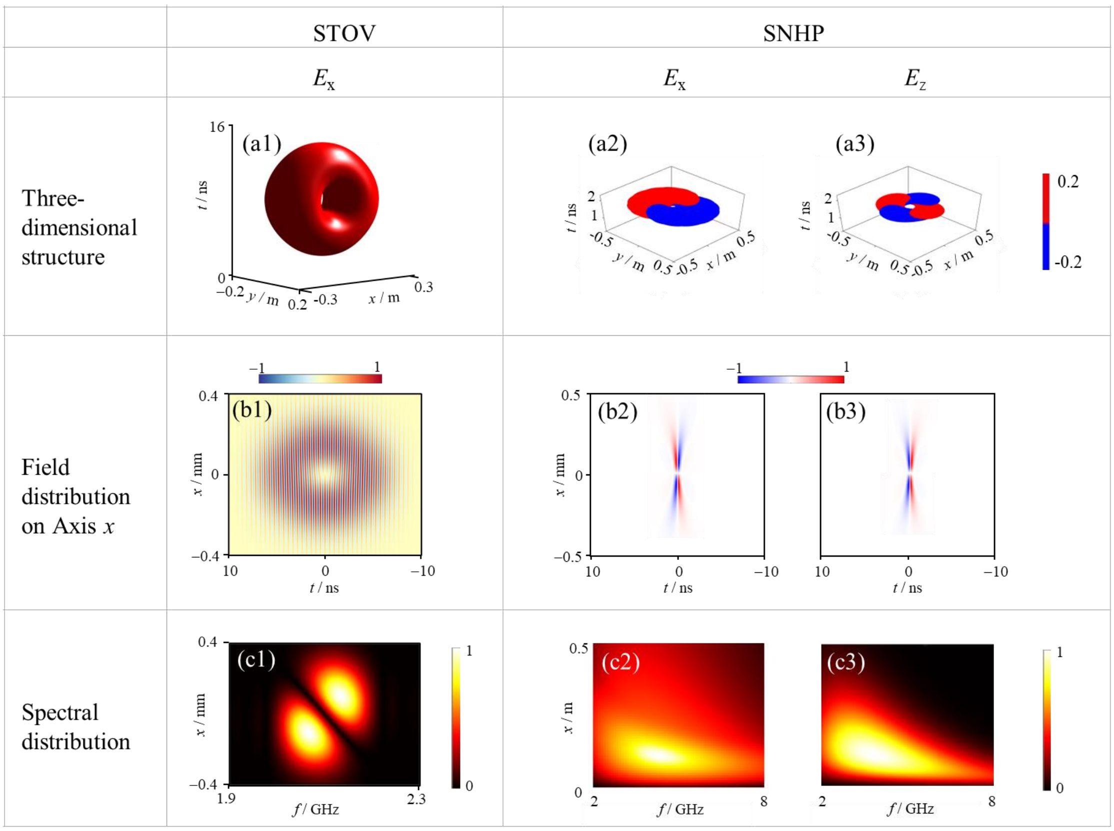

Supplementary Figure 32. A brief comparison between SNHPs and STOVs. (a1) Three-dimensional spatiotemporal field envelope of a STOV, exhibiting a toroidal topology with a nodal line perpendicular to the propagation direction. (a2), (a3) Three-dimensional spatiotemporal fields of the transversely and longitudinally polarized components of an SNHP, respectively, both showing helical topologies with nodal lines aligned parallel to the propagation direction. (b1) Spatiotemporal field distribution along the x-axis for a STOV, featuring a planar wavefront and a multi-cycle structure with a central singularity. (b2), (b3) Spatiotemporal field distributions along the x-axis for the transversely and longitudinally polarized components of an SNHP, respectively, both showing single-cycle profiles. (c1) Spatial–spectral distribution of a STOV with

~10% bandwidth, characterized by a tilted nodal line. (c2), (c3) Spatial–spectral distributions of the transversely and longitudinally polarized components of an SNHP, respectively, each with a bandwidth exceeding 120% and a triangular-shaped dominant spectral region.
